# Human G3P[4] rotavirus obtained in Japan, 2013, possibly emerged through a human–equine rotavirus reassortment event

**DOI:** 10.1007/s11262-014-1135-z

**Published:** 2014-10-29

**Authors:** Rungnapa Malasao, Mayuko Saito, Akira Suzuki, Toshifumi Imagawa, Nao Nukiwa-Soma, Kentaro Tohma, Xiaofang Liu, Michiko Okamoto, Natthawan Chaimongkol, Clyde Dapat, Kazuhisa Kawamura, Yasuko Kayama, Yoshifumi Masago, Tatsuo Omura, Hitoshi Oshitani

**Affiliations:** 1 Department of Virology, Tohoku University Graduate School of Medicine, Seiryo-cho 2-1, Aoba-ku, Sendai, Miyagi 980-8575 Japan; 2 Virus Research Center, Sendai Medical Center, Sendai, Japan; 3 Kawamura Pediatric Clinic, Sendai, Japan; 4 Kayama Pediatric Clinic, Sendai, Japan; 5 New Industry Creation Hatchery Center, Tohoku University, Sendai, Japan

**Keywords:** Rotavirus, G3P[4], Equine, Reassortment, Sendai

## Abstract

Two novel G3P[4] rotavirus strains were detected from children with acute diarrhea in Sendai, Japan, identified as a G3–P[4]–I2–R2–C2–M2–A2–N2–T2–E2–H2 genotype constellation by whole-genome sequence analysis. The VP7 gene of the two strains displayed the highest nucleotide sequence identity (91 %) and showed a close genetic relationship (99 % bootstrap value) to an equine rotavirus reported in India. The other gene segments were related to human group A rotaviruses. This report suggests a possible reassortment event between human and equine rotaviruses.

**Electronic supplementary material:**

The online version of this article (doi:10.1007/s11262-014-1135-z) contains supplementary material, which is available to authorized users.

Rotaviruses are a leading cause of acute diarrhea in young children and cause approximately 453,000 deaths per year worldwide [1]. In Japan, 9–23 % of children with acute gastroenteritis were infected by human group A rotaviruses (RVAs) [2–5].

RVAs belong to the family *Reoviridae*, genus *Rotavirus*. Its double-stranded RNA genome consists of 11 gene segments that encode six structural viral proteins (VP1–4, VP6–7) and six non-structural proteins (NSP1–6). The genes of the two antigenically distinct outer capsid proteins, VP7 (G type) and VP4 (P type), are used for genotyping [6]. To date, 27 G and 35 P genotypes have been identified based on the VP7 and VP4 sequences, respectively [7]. The five major combinations of G and P genotypes of human RVA circulating worldwide are G1P[8], G2P[4], G3P[8], G4P[8], and G9P[8] [8]. Moreover, G12 rotavirus is emerging globally [9–14]. A whole-genome classification system was developed to assign the genotype constellations as G*x*–P[*x*]–I*x*–R*x*–C*x*–M*x*–A*x*–N*x*–T*x*–E*x*–H*x* (where “*x*” depicts the number of the genotype) for symbolizing the VP7–VP4–VP6–VP1–VP2–VP3–NSP1–NSP2–NSP3–NSP4–NSP5 genes [15]. Two major and one minor representative genotype constellations of human RVAs, Wa-like (G1–P[8]–I1–R1–C1–M1–A1–N1–T1–E1–H1), DS-1-like (G2–P[4]–I2–R2–C2–M2–A2–N2–T2–E2–H2), and AU-1-like (G3–P[9]–I3–R3–C3–M3–A3–N3–T3–E3–H3), were reported to have RVA gene segments of porcine, bovine, and feline/canine origins, respectively [15]. The segmented double-stranded RNA genome of rotavirus facilitates reassortment among different strains [6, 16]. Genetic reassortment of rotaviruses can occur between human and human rotaviruses [17, 18], human and animal rotaviruses [19–21], or animal and animal rotaviruses [22].

According to human rotavirus epidemiology in Japan from 2003 to 2011, most common genotypes were G1P[8] followed by G3P[8], G9P[8], and G2P[4] [2–5]. Rotavirus vaccines were introduced in Japan in 2011 and the vaccine coverage between January and April 2013 was estimated to be 40–45 % [23]. During our acute gastroenteritis surveillance project conducted in Sendai, Japan, we detected two novel human G3P[4] rotaviruses which were detected rarely from children [9]. The objective of this study is to characterize these viruses by whole-genome sequencing and phylogenetic analysis.

Either rectal swabs or rapid test kit residues (BD Rota/Adeno Examen stick, Becton–Dickinson, New Jersey, USA) were collected from children with acute diarrhea in two pediatric clinics from November 2011 to April 2014. Samples were then transported on ice and viral RNA was extracted automatically using QIAcube (Qiagen, Hilden, Germany) with the QIAamp Viral RNA Mini QIAcube kit (Qiagen, Hilden, Germany). Complementary DNA was synthesized using SuperScript III reverse transcriptase (Invitrogen, Carlsbad, CA, USA). In the 10-μl reaction volume, 5 μl of viral RNA was mixed with 0.5 μl of 100 % DMSO [Final concentration (F.C): 5 %], and 0.5 μl of 300 ng/μl random hexamer primers (Invitrogen, Carlsbad, CA, USA) (F.C: 15 ng/μl) and 0.5 μl of 10 mM dNTP (F.C: 0.5 mM), heated at 98 ^°^C for 5 min, and cooled on ice. Then 2 μl of 5× first strand buffer (F.C: 1×), 0.5 μl of 0.1 M DTT (F.C: 5 mM), 0.25 μl of 40U/μl RNaseOUT (F.C: 1U/μl), and 0.25 μl of 200 U/μl superscript III RT (F.C: 5 U/μl) were added into the mixture. The reverse transcription step was carried out at 25 °C for 10 min, 50 °C for 50 min, followed by heating at 70 °C for 15 min to inactivate the enzyme, and cooling at 4 °C immediately. For screening of RVA, real-time PCR was performed [24] and for determining the G and P genotypes, conventional PCR was performed based on the amplified PCR products of partial VP7 and VP4 genes [25]. For sequencing of other genome segments of RVA, additional PCR was performed to amplify each genome segment based on the protocol published previously [26]. Nucleotide sequencing was performed using BigDye Terminator version 1.1 Cycle Sequencing kit and Genetic Analyzer 3730 (Applied Biosystems, Foster City, USA).

Basic Local Alignment Search Tool (BLAST) program was used to determine the sequence identity and similarity between query sequences and sequences available in GenBank. Phylogenetic trees based on the complete nucleotide sequences of each genome segment, which is 15–25 nucleotides at the termini of each genome segments not sequenced directly but fixed by the sequence of the primer used, were constructed using the neighbor-joining method of the MEGA5.2 program with 1,000 bootstrap replicates. To improve the calculation of genetic distances at the nucleotide level among sequences, the Kimura two-parameter nucleotide substitution model was used in inferring the phylogenetic trees [27]. The genotype of each segment was analyzed using the RotaC program (http://rotac.regatools.be/) [28]. The study protocol was approved by the ethics committee of Tohoku University Graduate School of Medicine.

The detection rate of RVA in the samples obtained from January to June 2013 at the two study clinics was 43.4 % (40/92). The most prevalent genotype was G1P[8] (22/40, 55.0 %). Interestingly, two samples were of the G3P[4] genotype. One of the samples (S13–30) was collected from a 4-year-old boy in the last week of February 2013 and the other (S13–45) was collected from a 1-year-old infant girl in the middle of March 2013. Both children did not receive rotavirus vaccines.

In Table 1, analysis of the complete genome sequences of these two strains revealed that the nucleotide sequence identity of the NSP5 gene between them was 100 %, while those for other segments were 99.2–99.9 %. The genotypic constellation of all 11 genome segments of both G3P[4] strains was G3–P[4]–I2–R2–C2–M2–A2–N2–T2–E2–H2. Both strains shared the same genotype constellation with human DS-1-like RVA strains except for the G genotype, which was G3. Comparison of the complete RVA gene segments among the two strains and other representative reference strains from the BLAST result revealed that ten gene segments other than G gene of both strains were related to human strains (97–99 % nucleotide identity) that were circulating in Australia, Europe, and Asia. The G gene, however, showed the highest nucleotide identity to an equine strain (Equine/IND/Erv105/G3) found in foal from India (91 %).

**Table 1:** Genotype of all 11 gene segments in two unusual G3P[4] rotavirus strains (S13–30 and S13–45)

Genotype (gene)	% nucleotide sequence identity between S13–30 and S13–45	Genotype constellation	Representative reference strains* which most closely related with our strains from BLAST result as of July 15, 2014 [Accession number] (% sequence identity comparing with our strains)
G (VP7)	99.9	G3	Equine/IND/Erv105/G3 [DQ981479] (91.0 %)
P (VP4)	99.9	P[4]	Hu/AUS/MP42/2010/G2P[4] [KF690136] (99.0 %)
I (VP6)	99.2	I2	Hu/THA/CMH030/2007/G2P[4] [JQ043294] (98.0–99.0 %)
R (VP1)	99.7	R2	Hu/AUS/CK20027/2006/G2P[4] [KC443763] (99.0 %)
C (VP2)	99.8	C2	Hu/JPN/HC12016/2012/G1P[8] [AB848008] (99.0 %)
M (VP3)	99.4	M2	Hu/JPN/HC12016/2012/G1P[8] [AB848009] (99.0 %)
A (NSP1)	99.6	A2	Hu/BGD/MMC88/2005/G2P[4] [HQ641368] (99.0 %)
N (NSP2)	99.6	N2	Hu/IDN/BL-5210/2006/G2P[4] [JQ837885] (99.0 %)
T (NSP3)	99.9	T2	Hu/ITA/PA3/2004/G2P[4] [KC178738] (98.0 %)
E (NSP4)	99.8	E2	Hu/AUS/RCH272/2012//G3P[14] [KF690134] (97.0 %)
H (NSP5/6)	100.0	H2	Hu/ITA/PA150/2006/G2P[4] [KC178757] (99.0 %)

*AUS* Australia, *THA* Thailand, *JPN* Japan, *BGD* Bangladesh, *IDN* Indonesia, and *ITA* Italy

* The reference strains carrying C2, M2, and E2 are reassortants themselves

Phylogenetic trees of all 11 segments were constructed based on complete nucleotide sequences of each gene (VP7 gene in Fig. 1 and others in supplementary material of S1–S10). The two novel strains, S13–30 and S13–45, belonging to G3 genotype, clustered with the equine Erv105 strain with a high bootstrap value of 99 %. The G3 genotype generally has a wide host range including human, simian, feline, canine, bovine, porcine, lapine, equine, murine, avian, and ovine species [7, 19, 21]. The P[4] genotype, on the other hand, has a narrow host range; over 95 % of RVA infections have been found in humans [29].

**Fig. 1:**
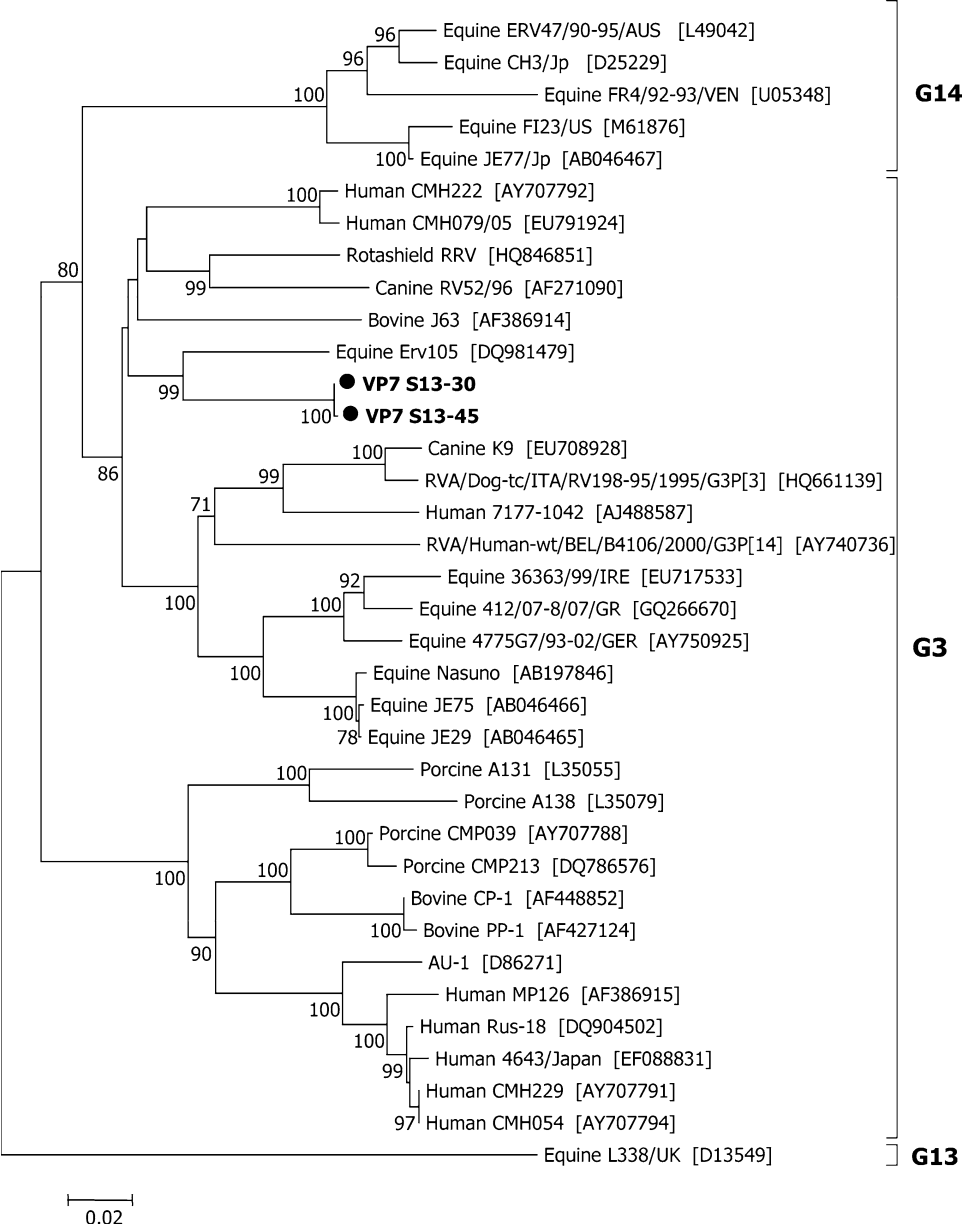
Phylogenetic analysis of the complete VP7 (G genotype) gene. The two unusual G3P[4] rotavirus strains (S13–30 and S13–45), which were detected in Sendai in 2013 are indicated in* boldface*. Bootstrap values (1,000 replicates) above 70 % are shown. The tree was constructed using the neighbor-joining with MEGA5.2 program. Accession numbers of reference strains defined by suffixes are shown in square brackets

Genetic reassortment between the RVAs of human and animals origins has been reported for human–porcine [30], human–simian [21], human–bovine [31], human–feline/canine [20], human–caprine [21], human–lapine [32], and human–ovine [33]. However, no report of genetic reassortment between human and equine rotavirus has been documented. The strong bootstrap support and the high nucleotide similarity between the VP7 genes of the two novel G3P[4] rotaviruses with an equine rotavirus suggest an equine origin of the human VP7 gene and provide evidence of a possible reassortment between human and equine rotaviruses.

Equine rotavirus is the leading cause of severe dehydrating diarrhea in foal aged up to 3 months in Europe, America, Australia, and Asia. The commonly reported G-P combinations of equine rotavirus are G3P[12] and G14P[12] genotypes [34]. Based on cross neutralization assays, G3 equine rotaviruses are further divided into 2 subtypes: the G3A subtype mostly circulating in Europe, America, and Australia, and the G3B subtype mostly circulating in Asia, especially in Japan [34]. In Asia, the information on equine rotavirus genotype is limited to Japan [35–37] and India [38]. The predominant G3 subtype of equine rotavirus circulating in Japan was G3B which was detected over a period of three decades (1981–2010) [34]. The G3 sequences of the two novel strains in our study were phylogenetically different from those identified in horses in Japan (Fig. 1). Unfortunately, no comprehensive analysis has been conducted for G3 equine rotaviruses in India. Based on the phylogenetic analysis of VP7 nucleotide sequence, G3 equine rotaviruses detected in India were grouped separately from the other equine rotaviruses found in the world [39].

The genotype constellation of the novel G3P[4] strains was similar to the DS-1-like group (G2–P[4]–I2–R2–C2–M2–A2–N2–T2–E2–H2) (Table 1). The P[4] genotype (based on the VP4 gene) of our strains was closely related to the P[4] genotype reported in humans in Asia and Australia (Fig. S4). The genotypes of the remaining nine segments showed close genetic relationships to human rotaviruses found in Asia, Australia, US, and Europe (Fig. S1–S3, S5–S10). These findings suggest a wide geographical distribution of the DS-1-like genotypes.

In conclusion, this report describes two novel G3P[4] rotavirus strains detected from children in Sendai, Japan with VP7 genes of likely equine origin. This study highlights the importance of rotavirus surveillance in animals to permit identification of interspecies transmission and reassortment events.

Nucleotide sequence accession numbers: The nucleotide sequences of strain S13–30 and S13–45 have been deposited in GenBank under the accession numbers KJ639012–KJ639022 and KJ639023–KJ639033, respectively.

## Electronic supplementary material

Below is the link to the electronic supplementary material.

Supplementary material 1 (PDF 253 kb)
