# Supplementary material for: Human G3P[4] rotavirus obtained in Japan, 2013, possibly emerged through a human–equine rotavirus reassortment event
Source: Virus Genes. 2014 Oct 29;50(1):129–33. doi: 10.1007/s11262-014-1135-z (PMC4349953; doi:10.1007/s11262-014-1135-z)
Supplement: Supplementary file 1 — Supplementary material 1 (PDF 253 kb) [file 11262_2014_1135_MOESM1_ESM.pdf]

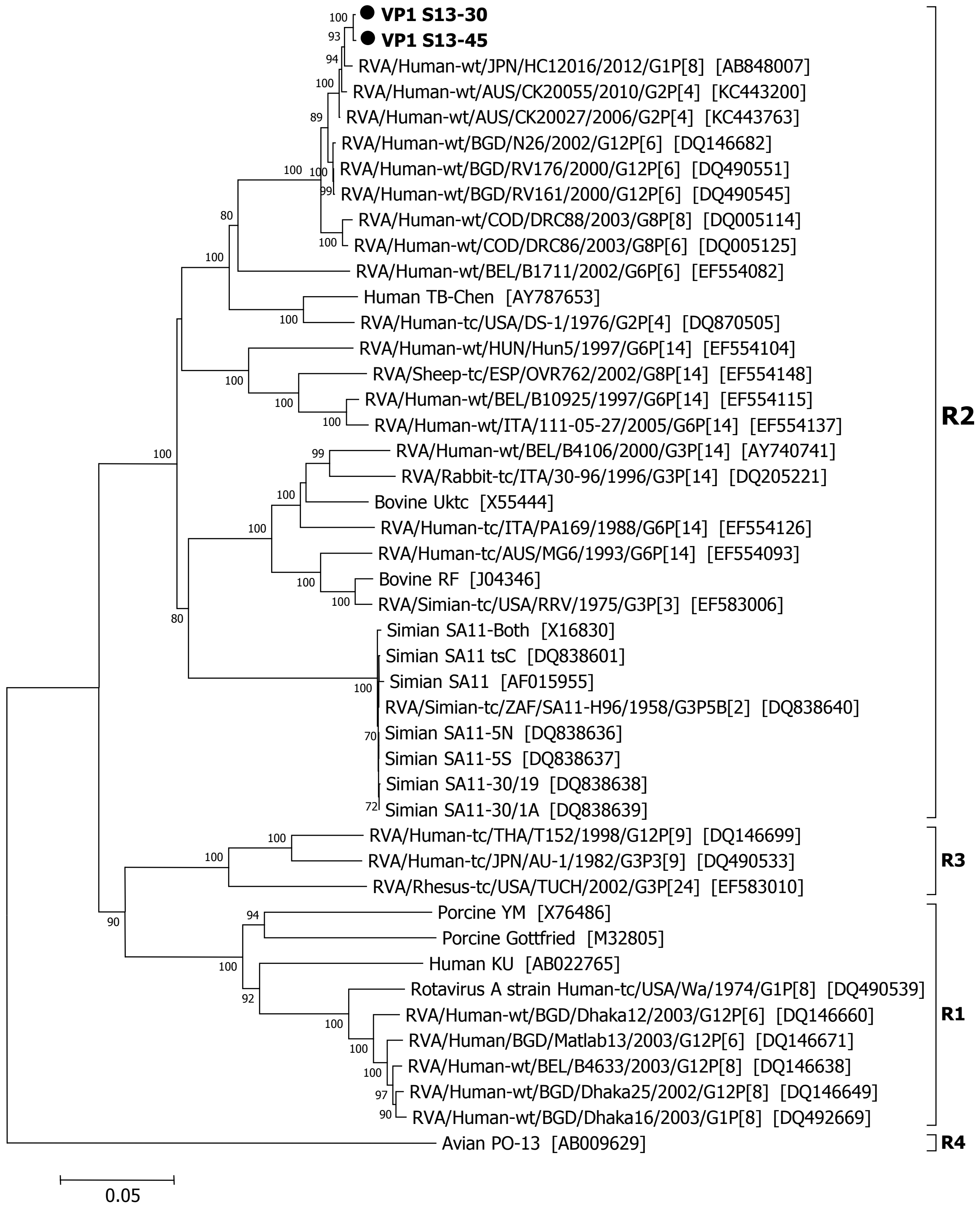

Fig. S1 Phylogenetic analysis of rotavirus whole VP1 (R genotype) nucleotide sequences. Both samples from Sendai, Japan belonged to genotype R2. Accession numbers of reference strains defined by suffixes were shown in square brackets.

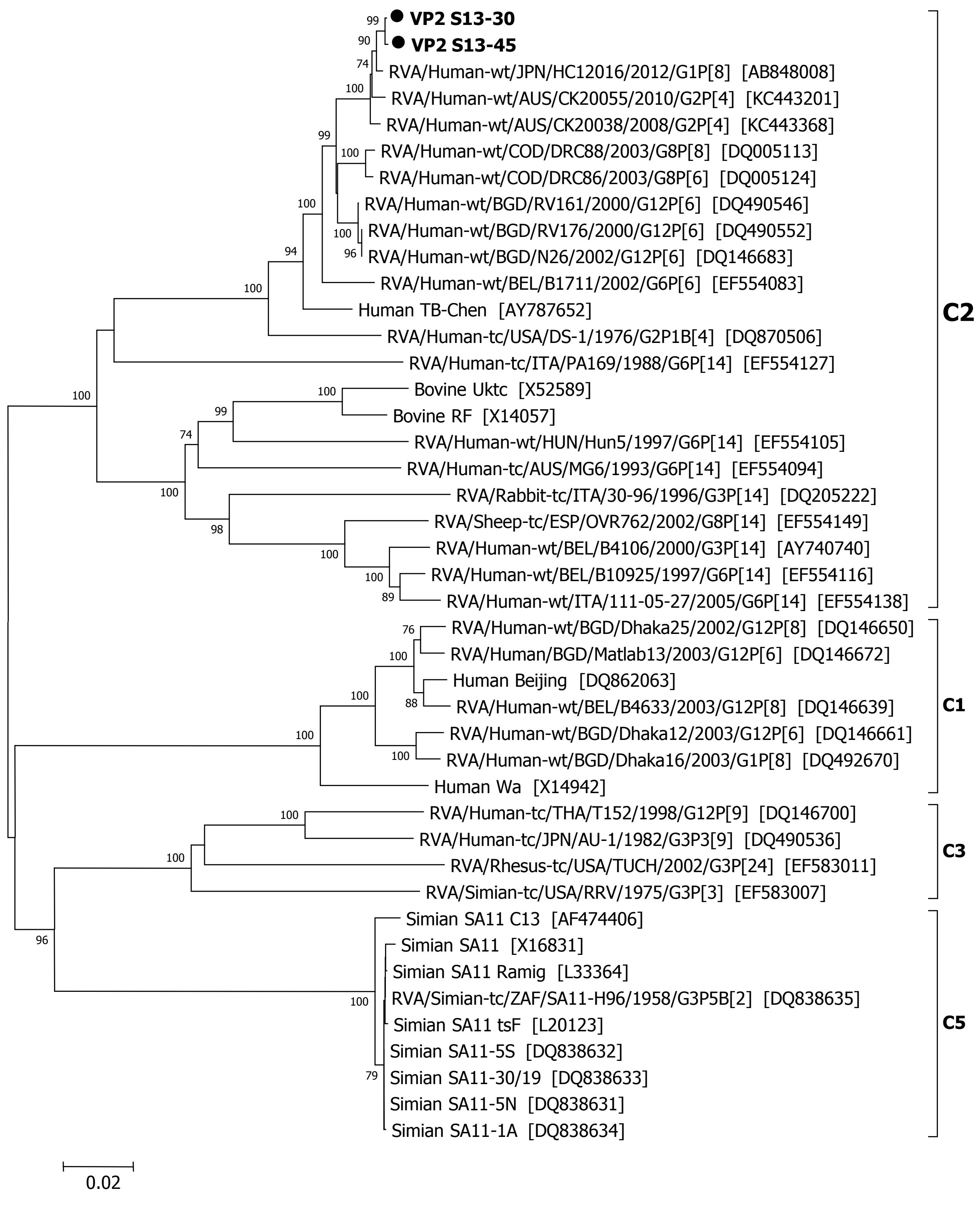

Fig. S2 Phylogenetic analysis of rotavirus whole VP2 (C genotype) nucleotide sequences. Both samples from Sendai, Japan belonged to genotype C2. Accession numbers of reference strains defined by suffixes were shown in square brackets.

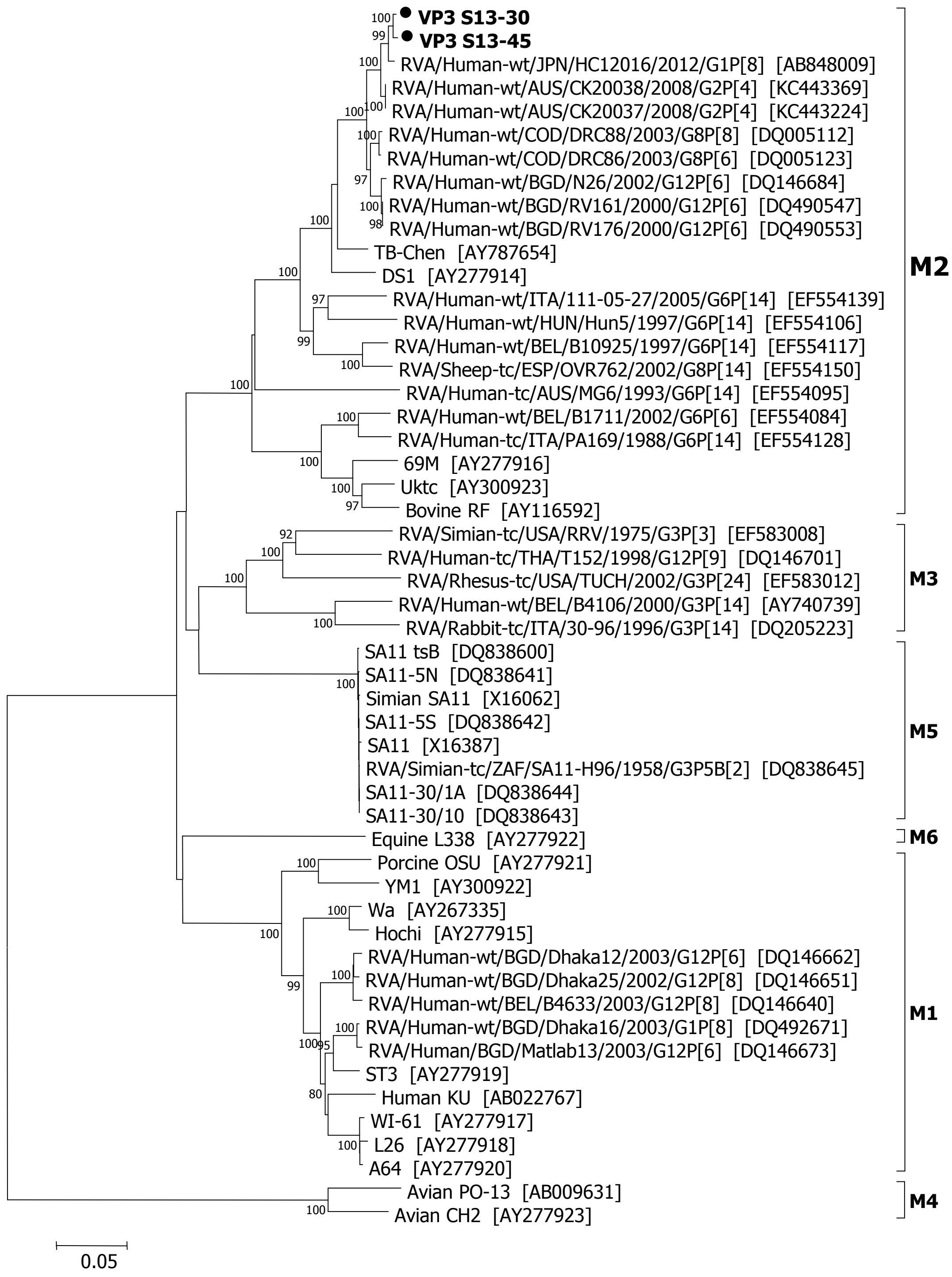

Fig. S3 Phylogenetic analysis of rotavirus whole VP3 (M genotype) nucleotide sequences. Both samples from Sendai, Japan belonged to genotype M2. Accession numbers of reference strains defined by suffixes were shown in square brackets.

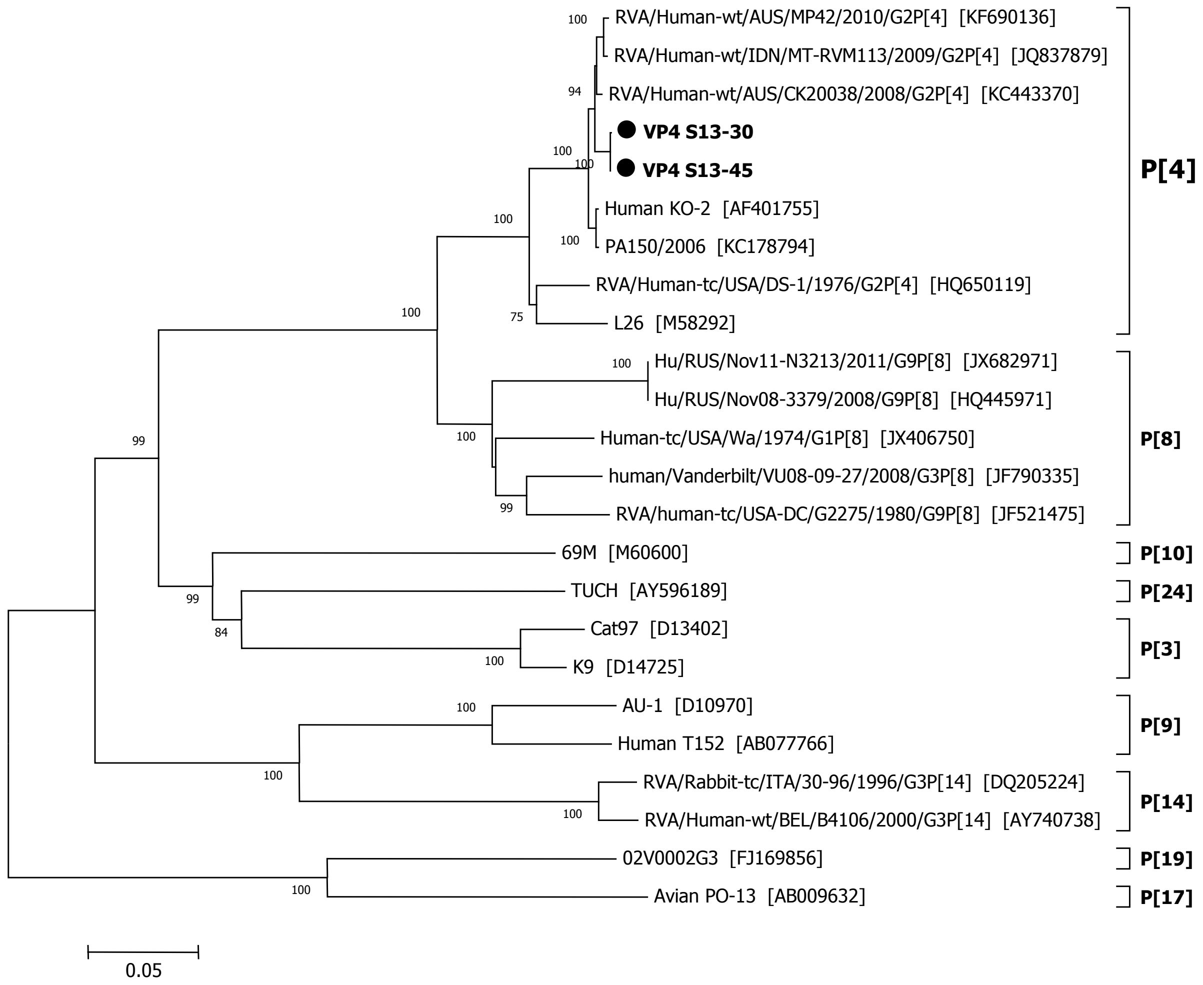

Fig. S4 Phylogenetic analysis of rotavirus whole VP4 (P genotype) nucleotide sequences. Both samples from Sendai, Japan belonged to genotype P[4]. Accession numbers of reference strains defined by suffixes were shown in square brackets.

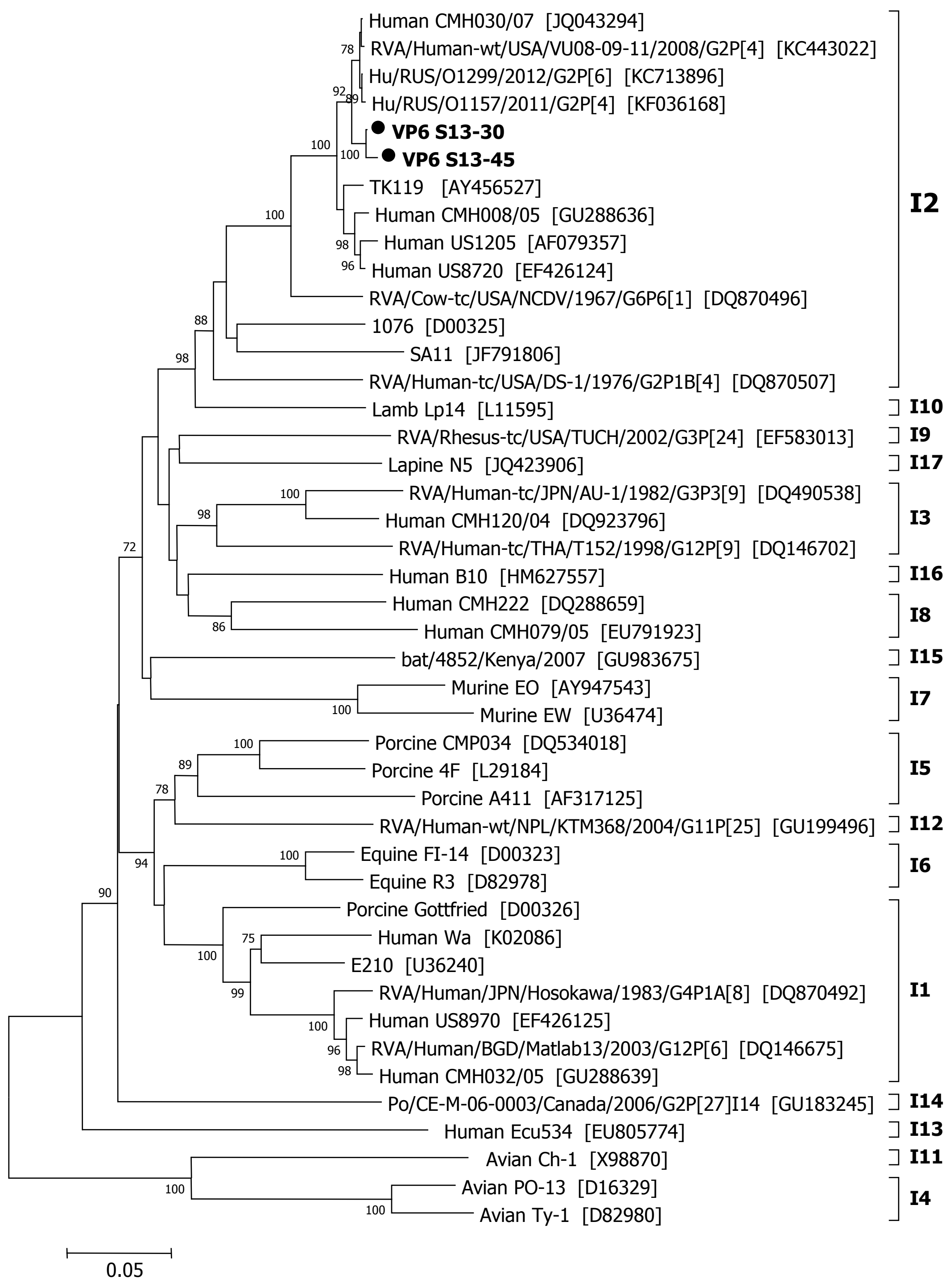

Fig. S5 Phylogenetic analysis of rotavirus whole VP6 (I genotype) nucleotide sequences. Both samples from Sendai, Japan belonged to genotype I2. Accession numbers of reference strains defined by suffixes were shown in square brackets.

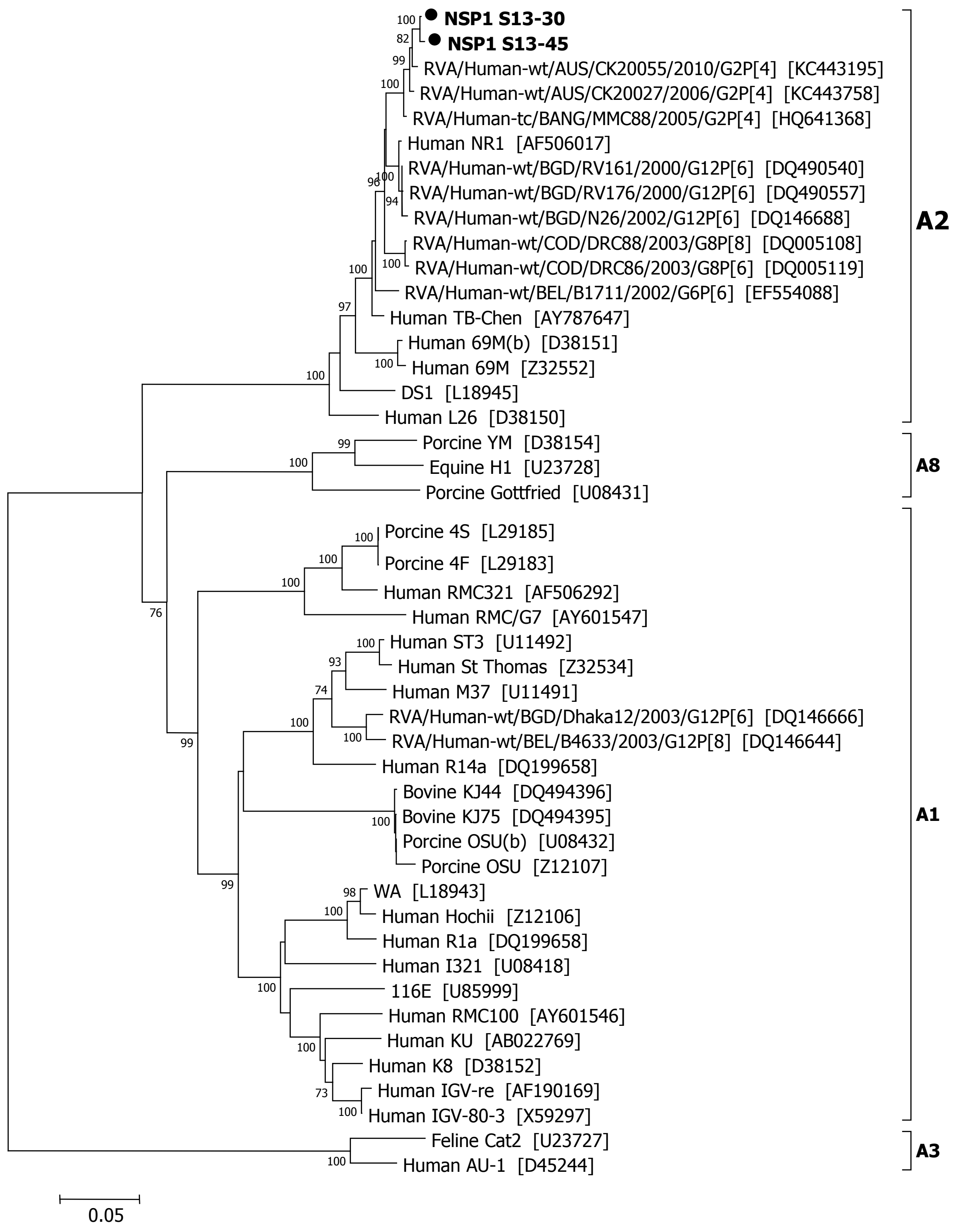

Fig. S6 Phylogenetic analysis of rotavirus whole NSP1 (A genotype) nucleotide sequences. Both samples from Sendai, Japan belonged to genotype A2. Accession numbers of reference strains defined by suffixes were shown in square brackets.

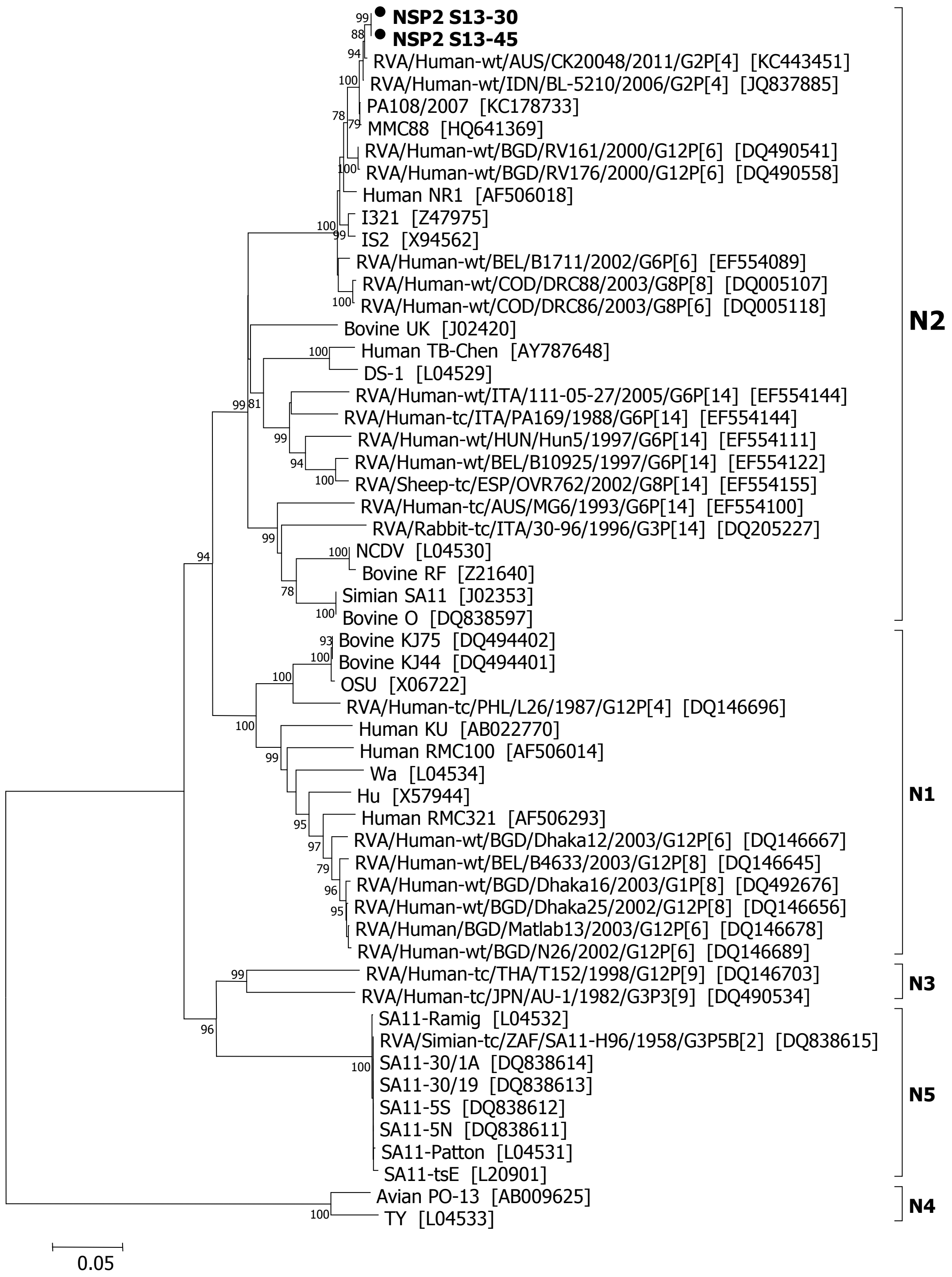

Fig. S7 Phylogenetic analysis of rotavirus whole NSP2 (N genotype) nucleotide sequences. Both samples from Sendai, Japan belonged to genotype N2. Accession numbers of reference strains defined by suffixes were shown in square brackets.

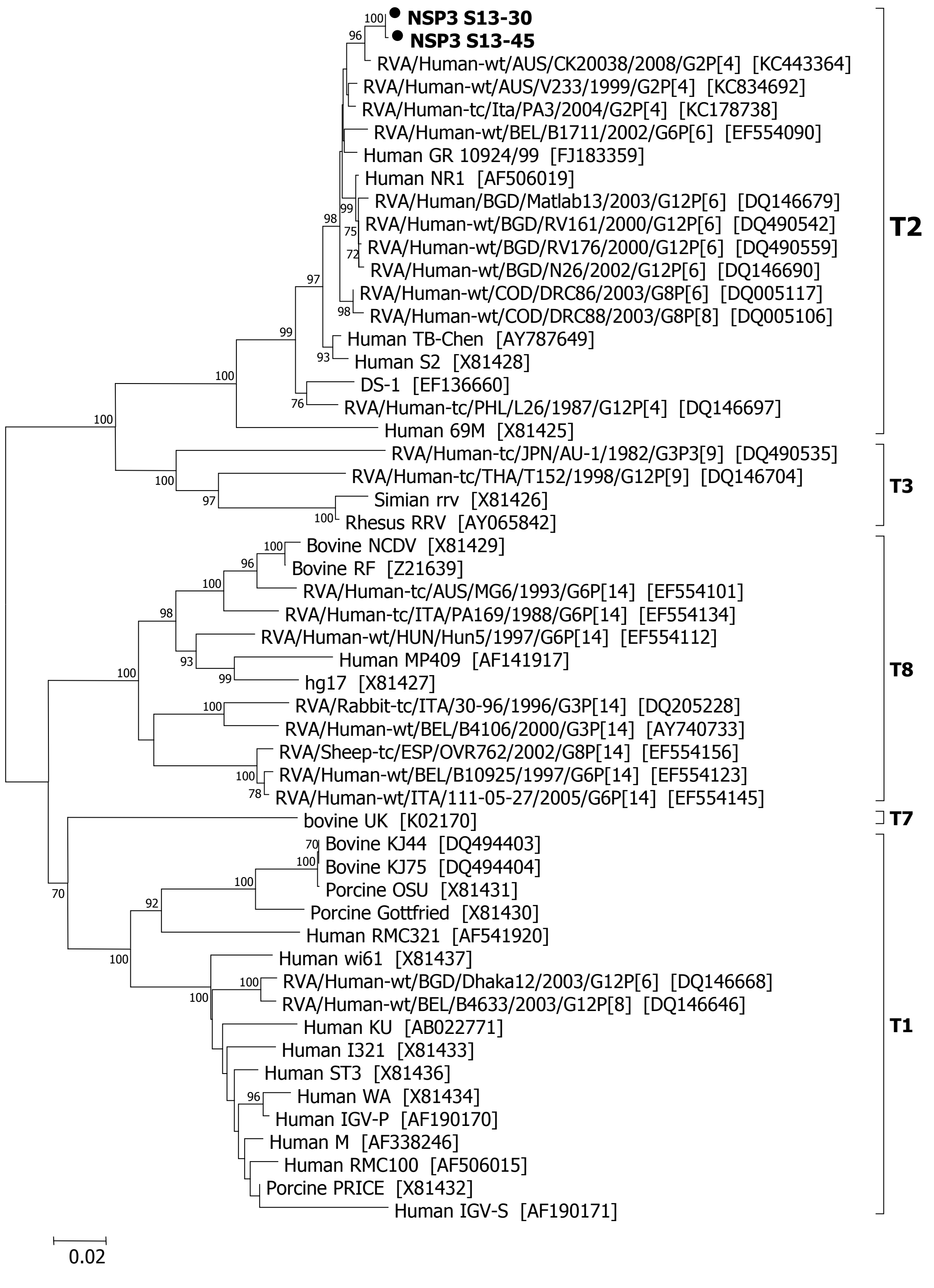

Fig. S8 Phylogenetic analysis of rotavirus whole NSP3 (T genotype) nucleotide sequences. Both samples from Sendai, Japan belonged to genotype T2. Accession numbers of reference strains defined by suffixes were shown in square brackets.

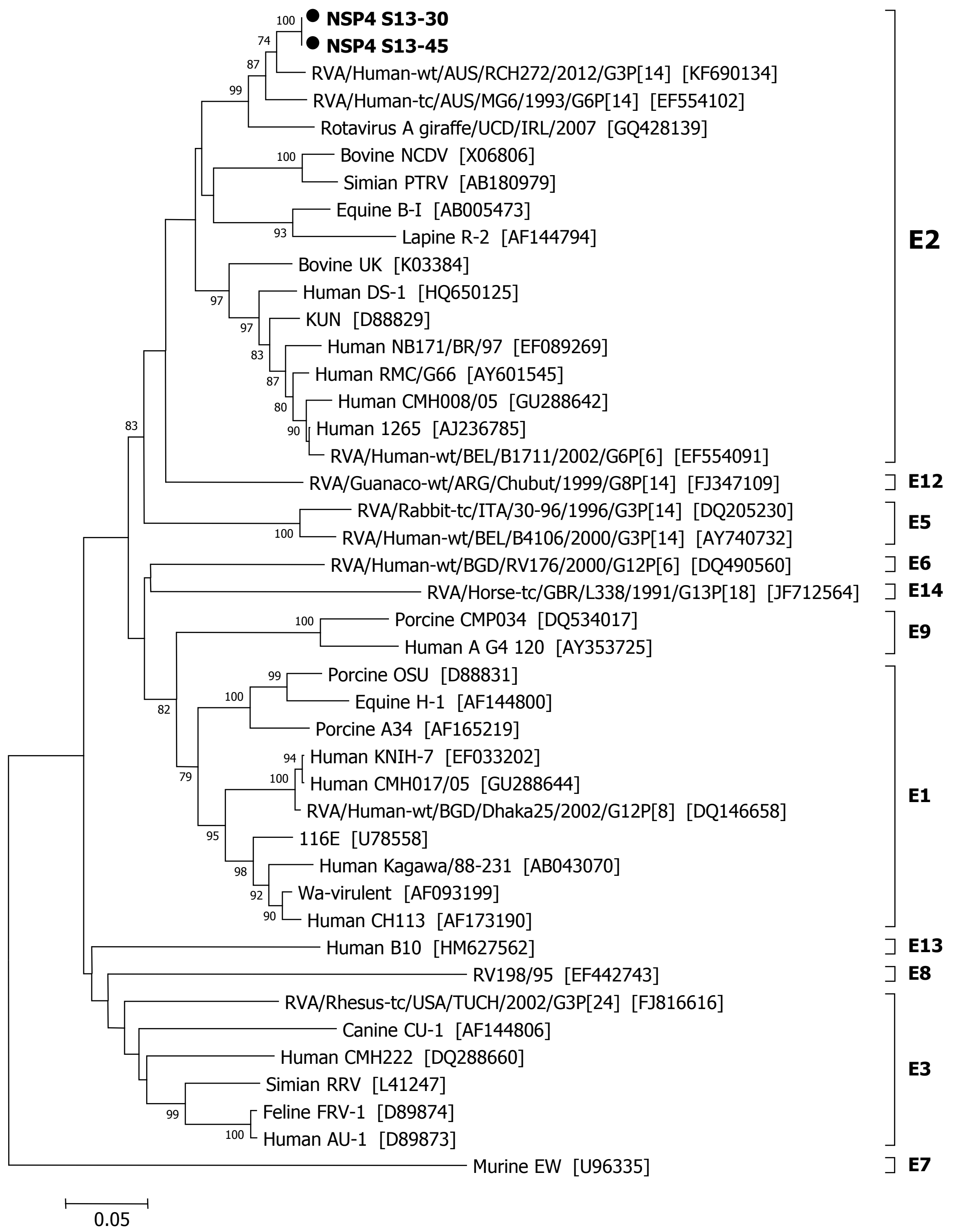

Fig. S9 Phylogenetic analysis of rotavirus whole NSP4 (E genotype) nucleotide sequences. Both samples from Sendai, Japan belonged to genotype E2. Accession numbers of reference strains defined by suffixes were shown in square brackets.

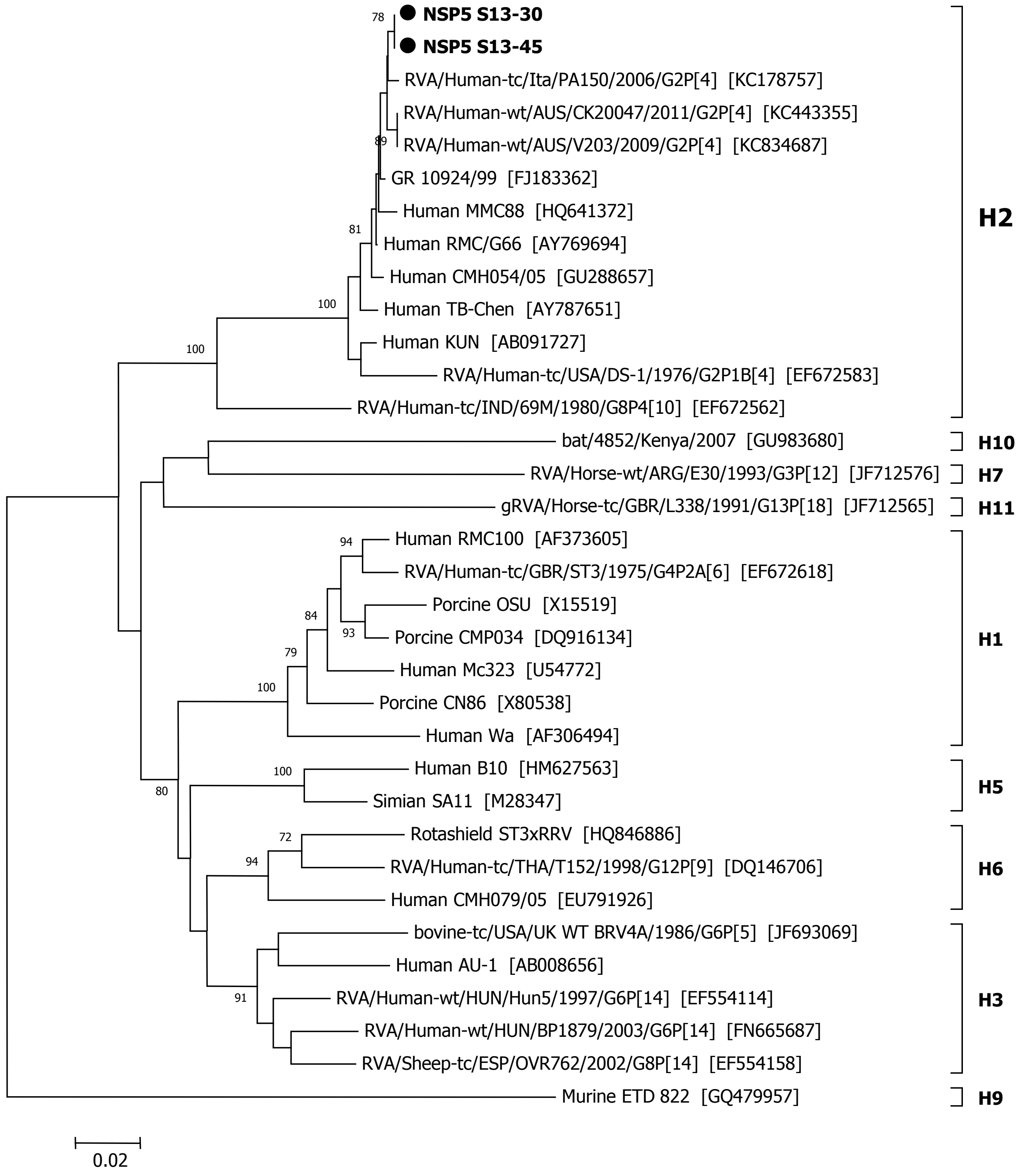

Fig. S10 Phylogenetic analysis of rotavirus whole NSP5/6 (H genotype) nucleotide sequences. Both samples from Sendai, Japan belonged to genotype H2. Accession numbers of reference strains defined by suffixes were shown in square brackets.
